# Ovarian stimulation with excessive FSH doses causes cumulus cell and oocyte dysfunction in small ovarian reserve heifers

**DOI:** 10.1093/molehr/gaad033

**Published:** 2023-09-15

**Authors:** Kaitlin R Karl, Peter Z Schall, Zaramasina L Clark, Meghan L Ruebel, Jose Cibelli, Robert J Tempelman, Keith E Latham, James J Ireland

**Affiliations:** Department of Animal Science, Reproductive and Developmental Sciences Program, Michigan State University, East Lansing, MI, USA; Department of Animal Science, Reproductive and Developmental Sciences Program, Michigan State University, East Lansing, MI, USA; Department of Animal Science, Reproductive and Developmental Sciences Program, Michigan State University, East Lansing, MI, USA; Department of Animal Science, Reproductive and Developmental Sciences Program, Michigan State University, East Lansing, MI, USA; Department of Animal Science, Reproductive and Developmental Sciences Program, Michigan State University, East Lansing, MI, USA; Department of Large Animal Clinical Sciences, Michigan State University, East Lansing, MI, USA; Department of Animal Science, Reproductive and Developmental Sciences Program, Michigan State University, East Lansing, MI, USA; Department of Animal Science, Reproductive and Developmental Sciences Program, Michigan State University, East Lansing, MI, USA; Department of Obstetrics, Gynecology and Reproductive Science, Michigan State University, East Lansing, MI, USA; Department of Animal Science, Reproductive and Developmental Sciences Program, Michigan State University, East Lansing, MI, USA

**Keywords:** small ovarian reserve, excessive FSH, superovulation, oocyte retrieval, cumulus–oocyte complexes, dysregulation of cumulus function, FSH target genes in cumulus cells, premature cumulus expansion, resumption of meiosis

## Abstract

Excessive FSH doses during ovarian stimulation in the small ovarian reserve heifer (SORH) cause premature cumulus expansion and follicular hyperstimulation dysgenesis (FHD) in nearly all ovulatory-size follicles with predicted disruptions in cell-signaling pathways in cumulus cells and oocytes (before ovulatory hCG stimulation). These observations support the hypothesis that excessive FSH dysregulates cumulus cell function and oocyte maturation. To test this hypothesis, we determined whether excessive FSH-induced differentially expressed genes (DEGs) in cumulus cells identified in our previously published transcriptome analysis were altered independent of extreme phenotypic differences observed amongst ovulatory-size follicles, and assessed predicted roles of these DEGs in cumulus and oocyte biology. We also determined if excessive FSH alters cumulus cell morphology, and oocyte nuclear maturation before (premature) or after an ovulatory hCG stimulus or during IVM. Excessive FSH doses increased expression of 17 cumulus DEGs with known roles in cumulus cell and oocyte functions (responsiveness to gonadotrophins, survival, expansion, and oocyte maturation). Excessive FSH also induced premature cumulus expansion and oocyte maturation but inhibited cumulus expansion and oocyte maturation post-hCG and diminished the ability of oocytes with prematurely expanded cumulus cells to undergo IVF or nuclear maturation during IVM. Ovarian stimulation with excessive FSH is concluded to disrupt cumulus cell and oocyte functions by inducing premature cumulus expansion and dysregulating oocyte maturation without an ovulatory hCG stimulus yielding poor-quality cumulus–oocyte complexes that may be incorrectly judged morphologically as suitable for IVF during ART.

## Introduction

Diagnosis of a small ovarian reserve (total number of morphologically healthy follicles/oocytes in ovaries) is the primary reason women seek ART (Centers for Disease Control and Prevention ASfRM, Society for Assisted Reproductive Technology, 2013). However, many women with small ovarian reserves respond poorly to treatments with FSH during ovarian stimulation protocols (Ireland *et al.*, 2011; Baker *et al.*, 2015; Clark *et al.*, 2021). Moreover, total FSH doses during ovarian stimulation protocols vary from <1000 to 20 000 IU (Baker *et al.*, 2015; Clark *et al.*, 2021), and high FSH doses during ovarian stimulation are inversely linked to ovarian function, oocyte recovery, and live birth rate during ART in women (Edwards *et al.*, 1996; Hazekamp *et al.*, 2000; Inge *et al.*, 2005; Klinkert *et al.*, 2005; Kovalevsky and Patrizio 2005; Pal *et al.*, 2008; Sterrenburg *et al.*, 2011; Baker *et al.*, 2015; Clark *et al.*, 2021) and ovarian function and embryo transfer outcomes in cattle (Greve *et al.*, 1979; Donaldson and Perry 1983; Donaldson 1984; McGowan *et al.*, 1985; Pawlyshyn *et al.*, 1986; Saumande and Chupin 1986; Sugano and Watanabe 1997). Whether excessive FSH doses during ovarian stimulation cause or contribute to poor ART outcomes, and the mechanisms responsible for such an effect, are unknown.

To better understand the impact of high FSH doses during ovarian stimulation on ovulatory follicle function, oocyte quality, and associated mechanisms, we developed the small ovarian reserve heifer (SORH) model. This unique model has biomedical relevance because it exhibits not only a relatively low number of morphologically healthy oocytes and a low antral follicle count (AFC) (Ireland *et al.*, 2007) but diminished circulating concentrations of anti-Müllerian hormone (Ireland *et al.*, 2008), progesterone and estradiol, hypersecretion of FSH (Abdalla and Thum 2004; Burns *et al.*, 2005; Ireland *et al.*, 2007) during reproductive cycles, and poor responsiveness to superovulation (Ireland *et al.*, 2007), like women with a small ovarian reserve.

Although recombinant FSH is not available for use in cattle, ovarian stimulation of the SORH model with different doses of Folltropin-V, a commercial FSH-enriched porcine pituitary preparation (cpFSH) with minimal LH contamination (<1%), has provided new insights into the potential detrimental impact of high FSH doses on ovulatory follicle function. For example, we established that cpFSH doses only 3-fold higher than the industry standard are excessive and thus economically wasteful because they do not increase number or size of ovulatory-size follicles compared with lower doses (Karl *et al.*, 2021). In addition, excessive cpFSH (Ex-cpFSH) doses are detrimental to ovulatory follicle function because they decrease both circulating estradiol concentrations and responsiveness of ovulatory-size follicles to hCG, thereby reducing ovulation rate (Karl *et al.*, 2021).

Using the SORH model, we also established that ovarian stimulation with the Ex-cpFSH doses results in premature cumulus cell expansion and luteinization (as measured by low intrafollicular estradiol but high progesterone and oxytocin concentrations) prior to an ovulatory hCG stimulus in a large proportion of the ovulatory-size follicles (Clark *et al.*, 2022a). Furthermore, based on whole transcriptome analysis data, we concluded that Ex-cpFSH doses during ovarian stimulation cause follicular hyperstimulation dysgenesis (FHD) in all ovulatory-size follicles (Clark *et al.*, 2022b). Severe abnormalities in multiple cell-signaling pathways in granulosa and cumulus cells and oocytes critical for folliculogenesis, steroidogenesis, luteinization, cell survival, ovulation, and oocyte maturation and quality characterize this disorder. Consequently, although the precise mechanisms remain unclear, FHD likely causes or contributes to the inhibition of ovulatory-size follicle growth, reduction in estradiol production, promotion of premature cumulus expansion and luteinization, and diminution of ovulation rate in response to hCG, as we observed during ovarian stimulation of the SORH model with Ex-cpFSH (Karl *et al.*, 2021; Clark *et al.*, 2022a,b). Our observations support the hypothesis that Ex-cpFSH doses during ovarian stimulation dysregulate cumulus function and oocyte maturation.

We tested our hypothesis here using the SORH model in two ways. First, we determined if Ex-cpFSH doses during ovarian stimulation resulted in dysregulation of cumulus cell genes critical for function and regulation of oocyte maturation. This was accomplished by manually interrogating our published whole transcriptome data set to determine whether any of the previously identified Ex-cpFSH-induced cumulus cell differentially expressed genes (DEGs) (Clark *et al.*, 2022b) are altered in all ovulatory-size follicles independent of individual follicular phenotypic differences, and whether these DEGs have known roles in cumulus cell function and oocyte maturation. We reasoned that cpFSH-induced overexpression of cumulus genes in all ovulatory-size follicles could explain the premature cumulus expansion observed in nearly all ovulatory-size follicles developing in response to Ex-cpFSH doses in our studies (Clark *et al.*, 2022a,b). Second, we tested whether the Ex-cpFSH-induced premature cumulus expansion observed in our studies (Clark *et al.*, 2022a,b) is accompanied by altered oocyte nuclear maturation before (premature) or after an ovulatory hCG stimulus, or during IVM.

## Materials and methods

### Analysis of Ex-cpFSH-induced differentially expressed cumulus genes and their predicted roles in regulating cumulus cell function and oocyte maturation

**Figure 1.**
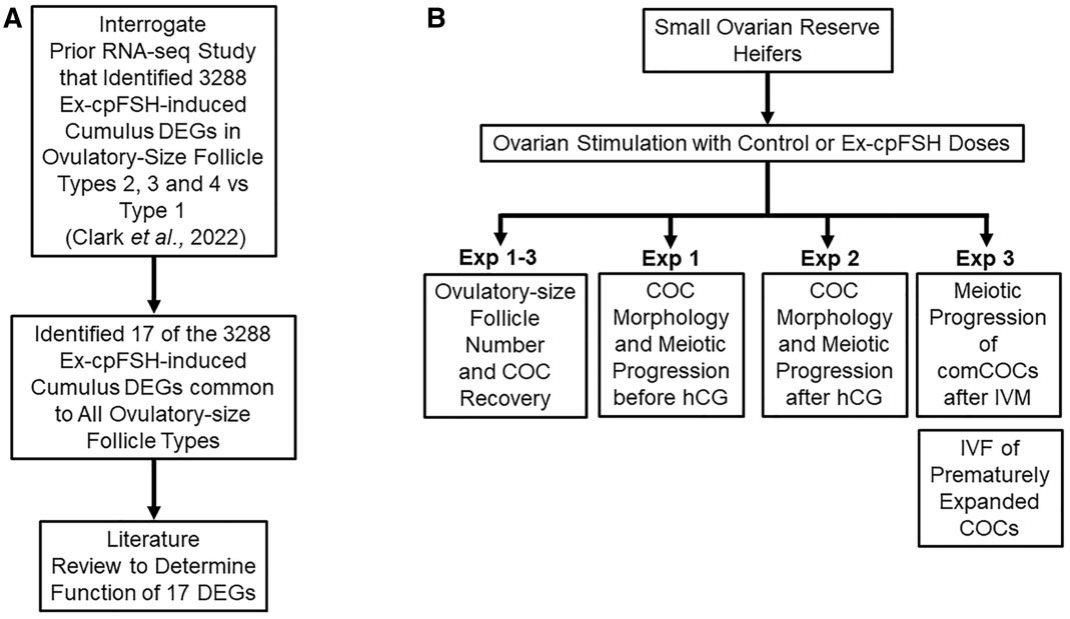
**Experimental approaches for analysis of FSH action on cumulus cell function and oocyte maturation in the small ovarian reserve heifer.** To test the hypothesis that excessive FSH action during ovarian stimulation dysregulates cumulus cell function and oocyte maturation, we determined: (**A**) if any of the 3288 Ex-cpFSH-induced cumulus DEGs in Types 2, 3, and 4 versus 1 ovulatory-size follicles of the SORH model, identified in our previously published transcriptome data set (Clark *et al.*, 2022b), were common to all ovulatory-size follicles independent of their Type and if these DEGs were critical for cumulus function and oocyte maturation. We also determined: (**B**) if Ex-cpFSH doses during ovarian stimulation of the SORH model altered number of ovulatory-size follicles and COC recovery rate (Exp 1–3), cumulus cell morphology (Exp 1–2), nuclear maturation of oocytes before (premature, Exp 1) or after an ovulatory hCG stimulus (Exp 2) or after IVM of comCOCs (Exp 3), and IVF of prematurely expCOCs (Exp 3). Ex-cpFSH, excessive commercial FSH-enriched porcine pituitary preparation; DEGs, differentially expressed genes; SORH, small ovarian reserve heifer; COC, cumulus–oocyte complex; Exp, experiment; comCOCs, compact cumulus–oocyte complexes; expCOCs, expanded cumulus–oocyte complexes.

In our previously published study (Clark *et al.*, 2022b), we analyzed by RNA sequencing four ovulatory-size follicle Types that had been identified based on FSH dose, cumulus–oocyte complex (COC) morphology (expanded, compact), and differences in intrafollicular concentrations of estradiol, progesterone and oxytocin (Clark *et al.*, 2022b). Type 1 ovulatory-size follicles were those from animals receiving the industry standard dose and mimic healthy ovulatory follicles during estrous cycles in cattle (Ireland and Roche 1982, 1983), having compact COCs (comCOCs), higher intrafollicular estradiol than progesterone concentrations, and relatively low oxytocin concentrations (Clark *et al.*, 2022a,b). Types 2, 3, and 4 follicles were identified in Ex-cpFSH treated SORH (Clark *et al.*, 2022a,b). Type 2 phenotypically resembles Type 1. Type 3 is like Type 1 and 2 but has an expanded layer of cumulus cells (expCOCs). Type 4 also has expCOCs but has much higher progesterone than estradiol and the highest oxytocin concentrations. Through RNA sequencing, we identified 4576 DEGs (characteristic of the FHD phenotype) in granulosa and cumulus cells and oocytes of the Ex-cpFSH-induced Type 2, 3, or 4 compared with the industry-standard cpFSH-induced Type 1 (control) ovulatory-size follicles. Of these 4576 DEGs, 3288 were observed in cumulus cells of the Type 2, 3, or 4 ovulatory-size follicles (Clark *et al.*, 2022b). In the present study (Fig. 1A), we manually interrogated each of these cumulus cell DEGs to identify those that were common to all three Ex-cpFSH ovulatory-size follicle types (Type 2, 3, and 4) and determined, based on a literature search, whether these DEGs have known roles in regulation of cumulus function and oocyte maturation (Fig. 1A).

### Effect of Ex-cpFSH on cumulus expansion and oocyte maturation

We conducted three experiments using the SORH model (Fig. 1B). The first experiment (Exp 1, Fig. 1B) determined whether Ex-cpFSH doses during ovarian stimulation altered cumulus morphology and stage or timing of oocyte nuclear maturation. Each ovulatory-size (≥10 mm in diameter) follicle for each heifer (n = 10) was subjected to oocyte retrieval 12 h after the last cpFSH dose (*no hCG stimulus given*) and each COC was classified morphologically as comCOCs or expCOCs, and stage of nuclear maturation attained for oocytes within each COC was determined. Exp 2 (Fig. 1B) determined whether Ex-cpFSH treatment during ovarian stimulation altered the capacity of cumulus cells and oocytes in ovulatory-size follicles to respond to an ovulatory hCG stimulus. Heifers were injected with an ovulatory dose of hCG 12 h after the last cpFSH injection. Each ovulatory-size follicle for each heifer (n = 16) was subjected to oocyte retrieval 24 h post-hCG but prior to ovulation. The responsiveness of cumulus cells and oocytes to the ovulatory hCG stimulus was then evaluated by morphological classification of each COC as comCOCs or expCOCs, and determination of nuclear maturation stage attained for the oocyte within each COC. Exp 3 (Fig. 1B) determined if the Ex-cpFSH doses during ovarian stimulation altered the capacity of expCOCs to resume meiosis during IVM and their ability to be fertilized by IVF. Each ovulatory-size follicle for each heifer (n = 18) was subjected to oocyte retrieval 12 h after the last cpFSH (*no ovulatory hCG stimulus given*) and each recovered COC was classified morphologically as comCOCs or expCOCs. The expCOCs from the Ex-cpFSH treated heifers (n = 8) were subjected to IVF and compared to comCOCs from abattoir ovaries that were also subjected to IVF. Additionally, a subset of Ex-cpFSH treated heifers (n = 10) was used to assess nuclear maturation stages of expCOCs versus comCOCs after IVM (Fig. 1B).

### Reagents

Unless otherwise mentioned, chemicals and reagents were purchased from Merck KGaA (Darmstadt, Germany).

### Identification of heifers with a small ovarian reserve

We previously established that 11- to 12-month-old heifers with a low AFC (≤15 follicles of ≥3 mm in diameter) during ovarian follicular waves (15–20% of a herd) also have 80% smaller ovarian reserves (total number morphologically healthy follicles and oocytes in ovaries) compared with age-matched counterparts with a high AFC (≥25 follicles) (Burns *et al.*, 2005; Ireland *et al.*, 2007, 2008, 2009; Jimenez-Krassel *et al.*, 2009; Mossa *et al.*, 2010). In the present study, serial ovarian ultrasonography was used to identify 11- to 12-month-old Holstein heifers of similar weights with a low AFC and small ovarian reserve (Karl *et al.*, 2021) for ovarian stimulation.

### Ovarian stimulation protocol

To synchronize estrous cycles for ovarian stimulation, heifers received an initial 2 ml i.m. injection of prostaglandin-F_2α_ (PG, 12.5 mg dinoprost/ml, Lutalyse *HighCon*, Zoetis, Parsipanny, NJ, USA) followed by two additional PG injections 12 h apart 10 days later. Each heifer underwent daily ovarian ultrasonography to detect ovulation and emergence of the first follicular wave. The first injection of Folltropin-V (porcine pituitary extract containing primarily FSH with 0.25% LH contamination (cpFSH), Vetoquinol USA Inc., Fort Worth, TX, USA) began 36 h after the last PG injection which was ±1 day from ovulation and initiation of the first follicular wave in all heifers. Heifers received 8 i.m. injections of cpFSH (either 70 IU or 210 IU) at 12-h intervals. The cpFSH dose range per injection was 20% lower and 240% higher than the Vetoquinol recommended dose per injection of 87.5 IU. Hereafter, the 70 IU industry-standard cpFSH dose is referred to as the control while the 210 IU cpFSH is referred to as the Ex-cpFSH dose. Following superovulation, either two or three i.m. PG injections were given 12 h apart starting at the time of the seventh cpFSH injection (about Day 4 or 5 of the estrous cycle) to regress the newly formed corpus luteum during each ovarian stimulation regimen. Oocyte retrieval was then conducted 12 h after the last cpFSH and PG injections or 24 h after a single 2.5 ml (2500 IU) i.m. injection of hCG (Chorulon HCG 10 000 IU, Merck Animal Health USA, Rahway, NJ, USA) to induce oocyte maturation but prior to ovulation.

### Oocyte retrieval from ovulatory-size follicles

For oocyte retrieval, heifers received caudal epidural anesthesia with lidocaine hydrochloride 2% (0.22 mg/kg; Lidocaine 2%, VetOne, Boise, ID, USA) mixed with xylazine hydrochloride 10% (0.025 mg/kg; AnaSed, VetOne) to minimize the stress of rectally manipulating ovaries for oocyte retrieval. Follicles ≥10 mm were aspirated using a real-time B-mode ultrasound scanner (Ibex EVO; E.I. Medical Imaging, Loveland, CO, USA) equipped with an 8.0 MHz microconvex transducer housed in a plastic vaginal probe with a stainless-steel needle guide connected to the aspiration equipment. COCs were aspirated from each follicle using an 18-gauge × 3-inch disposable follicular aspiration needle (Partnar Animal Health, Port Huron, MI, USA) connected to a Brazilian-style IVF tubing (Partnar Animal Health) and inserted into the stainless-steel needle guide. The contents of each follicle were aspirated into a 50 ml conical collection tube using an electric suction pump (K-MAR-5200, Cook Medical, Brisbane, Australia) at a variable negative pressure of 200 ± 1 mm Hg. Each 50 ml conical collection tube contained ∼3 ml of medium, which consisted of protein-free chemically defined hamster embryo culture medium-6 (HECM (Schramm and Al-Sharhan, 1996)) and HEPES. The HECM–HEPES (HH) medium (Patel *et al.*, 2007) was supplemented with 0.3% bovine serum albumin (BSA), 500 µM 3-isobutyl-1-methylxanthine (IBMX), and 100 nM C-type natriuretic peptide (NPPC). IBMX and NPPC were used to block oocyte maturation (Soto-Heras *et al.*, 2019). Before each oocyte retrieval, the needle, IVF tubing, and 50 ml conical collection tubes were pre-coated with HH medium supplemented with 0.3% BSA and 5% polyvinyl propylene.

### Morphological classification of COCs

To classify COCs, the contents of each 50 ml conical collection tube containing COCs were poured over an embryo filter (Hy-flow filter, SPI™, Canton, TX, USA) to isolate the COCs from each heifer. The filter was sprayed with HH medium to transfer COCs into a Petri dish. A stereomicroscope was used to classify each COC based on the number of cumulus cell layers (Yang *et al.*, 2017), as reported by us for cattle subjected to ovarian stimulation (Clark *et al.*, 2022a). comCOCs had one or more layers of cumulus cells surrounding the zona pellucida and oocyte whereas expCOCs had partially or totally expanded cumulus cells surrounding the zona pellucida and oocyte. Denuded oocytes devoid of cumulus cells were also recorded.

### IVM

When comCOCs or expCOCs were subjected to IVM, they underwent four washes in drops of HH medium without IBMX and NPPC followed by four washes in the IVM medium. The IVM medium consisted of Medium 199 supplemented with 22 µg/ml sodium pyruvate, 4 IU/ml hCG (Chorulon^®^; Merck & Co., Inc., Rahway, NJ, USA), 50 µg/ml gentamicin, and 100 µl/ml fetal bovine serum (Patel *et al.*, 2007). COCs were cultured in 10 µl drops of IVM medium in groups of 3–5 for 22 h at 38.5°C in a 5% CO_2_ atmosphere with 100% humidity.

To assess the nuclear maturation of COCs after IVM, our statistical power analysis limited us to groups of 3–5 COCs per drop from each heifer unless otherwise specified. However, a preliminary study was conducted to aspirate COCs from ovaries collected from cattle at a local abattoir (West Michigan Beef Co LLC, Hudsonville, MI, USA) to determine if 3 or 5 COCs per drop was representative of the proportion of oocytes reaching metaphase II (MII) during IVM. Chi square analysis indicated that groups of 3 (n = 55) or 5 (n = 31) COCs per drop did indeed produce similar proportions of MII oocytes during IVM (mean ± SEM, 71 ± 2% versus 80 ± 6%, respectively, *P* ≥ 0.85). Data for heifers that had <3 COCs recovered by oocyte retrieval were not subjected to IVM and were omitted from statistical analysis.

### IVF of expCOCs

Unless specified, all IVF media were purchased from IVF Bioscience, Cornwall, UK. The expCOCs were incubated at 5% CO_2_ in air at 38.5°C in high humidity. All expCOCs were rinsed in HEPES-buffered HECM containing 1 mg/ml BSA (HH-BSA) (Seshagiri and Bavister, 1989) and placed in the incubator in a 44 µl drop of pre-equilibrated BO-IVF medium under mineral oil. Frozen sperm from a single high fertility bull (Lolo, STgenetics, Navasota, TX, USA) was thawed at 38°C for 1 min and rinsed twice in BO-SEMENPREP medium using two consecutive 4 min centrifugations at 350*g* at 22°C. After the final rinse, the sperm pellet was resuspended in BO-IVF at a final concentration of 2.0 × 10^6^ sperm/ml in a 6 µl volume of the suspension, which was then added to 44 µl drops containing the expCOCs for 18 h. Subsequently, presumptive zygotes had their cumulus cells removed in 1 mg/ml hyaluronidase in HH-BSA using a 140 µm internal diameter glass pipette. After three rinses in HH-BSA, embryos were incubated in BO-IVC medium for 7.5 days at 38.5°C.

### Classification of stages of nuclear maturation

To determine nuclear maturation stages, oocytes were processed as reported (Prentice-Biensch *et al.*, 2012). The comCOCs and expCOCs were kept separate and washed three times in HH medium containing IBMX and NPPC and denuded by placing them into 2 ml microcentrifuge tubes containing 200 µl of 1% hyaluronidase solution at 37°C for 5 min followed by vortexing for 5 min. Denuded oocytes were then washed three times in HH medium containing IBMX and NPPC, transferred to four-well plates, and incubated for 15 min at room temperature in a permeabilization and fixation medium (6% Triton X-100 (v:v) in 3.7% paraformaldehyde solution (wt/vol)). The oocytes were washed three times again in Dulbecco’s PBS without calcium and magnesium for 15 min at room temperature. Groups of ≤5 oocytes from comCOCs or expCOCs were transferred to 5 µl drops of VectaShield^®^ Plus Antifade Mounting Medium with 4',6-diamidine-2'-phenylindole dihydrochloride (DAPI; Vector Laboratories^®^, Inc., Burlingame, CA, USA, H-2000-10) and mounted onto a glass slide under a coverslip.

**Figure 2.**
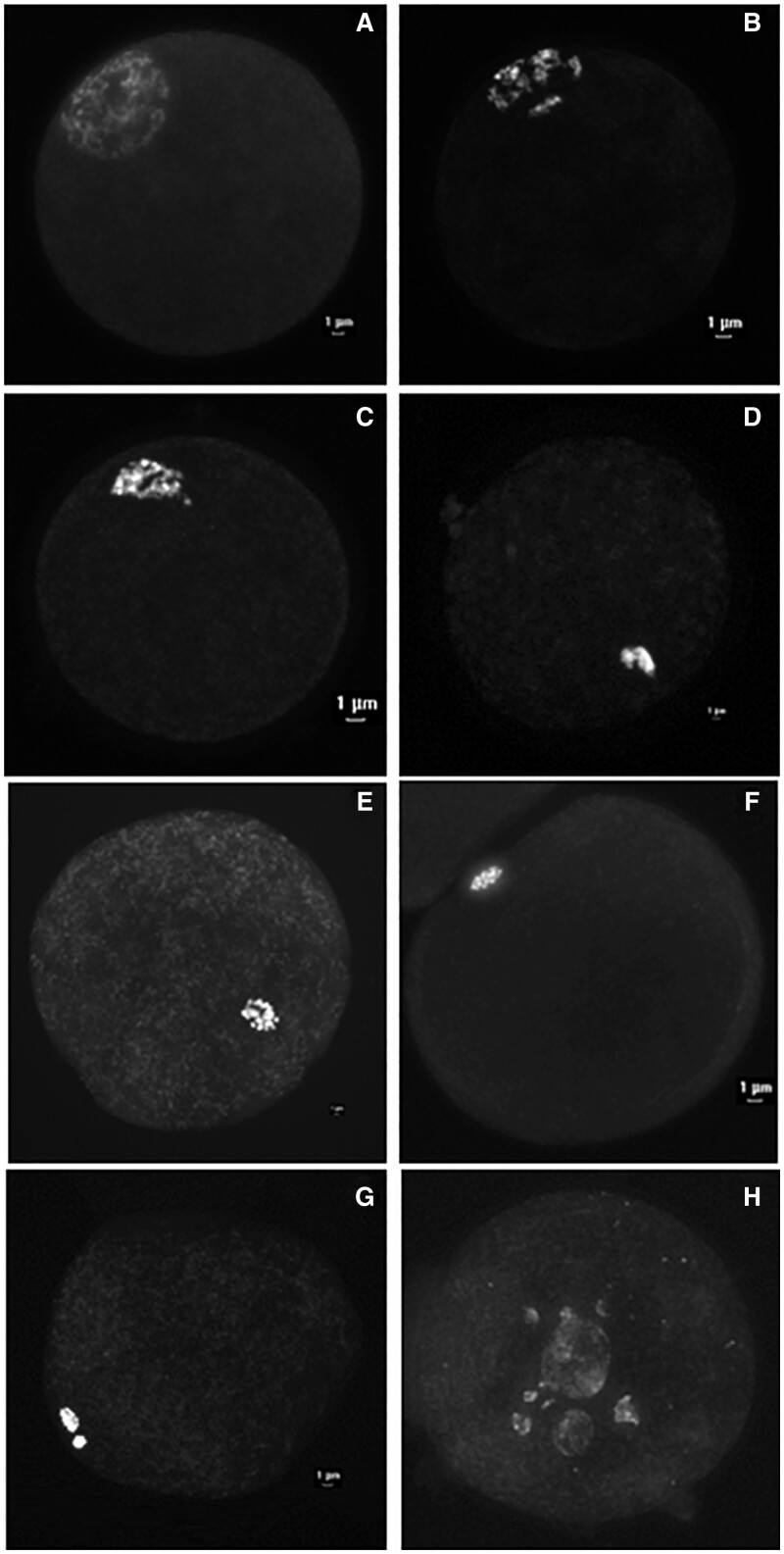
**Representative images of the different stages of nuclear maturation of oocytes from the in the small ovarian reserve heifer.** COCs were denuded, stained with DAPI and oocyte nuclear maturation stage determined as explained in Materials and Methods. The GV stages of nuclear maturation of oocytes are as follows: (**A**) GV0 = diffuse filamentous pattern of chromatin in the whole nuclear area, occasionally surrounding the nonfluorescent nucleolus; (**B**) GV1 = similar to GV0 except only a few condensed chromatin foci were detected in the nucleus; (**C**) GV2 = chromatin was further condensed into clumps, or a strand distributed throughout the nucleoplasm; or (**D**) GV3 = chromatin condensed into a single clump within the nuclear envelope. Oocytes not classified at a GV stage were classified as: (**E**) germinal vesicle breakdown = GVBD, (**F**) metaphase I = MI, (**G**) metaphase II = MII, or (**H**) degenerated = oocytes with fragmented chromosomes or without visible nuclear material. COCs, cumulus–oocyte complexes; GV, germinal vesicle.

Oocytes were examined under an epifluorescence microscope to determine the stage of nuclear maturation (Lodde *et al.*, 2007; Prentice-Biensch *et al.*, 2012) (Fig. 2). Oocytes with intact germinal vesicles (GV) were classified as G0, G1, G2, and G3 as depicted (Fig. 2A–D, respectively). The GV classifications were confirmed with anti-lamin A/C labeling, as reported (Prentice-Biensch *et al.*, 2012) (data not shown). The proportions of ovulatory-size follicles at the G0, G1, G2, or G3 nuclear stage were similar (*P* > 0.05) independent of cpFSH dose (data not shown), and thus combined into a single nuclear stage, hereafter called GV. All other oocytes not in the GV stage were classified as GV breakdown (GVBD), metaphase I (MI), or metaphase II (MII), as depicted (Fig. 2E–G, respectively). In addition, oocytes with no visible nuclear material or fragmented chromosomes were classified as degenerated.

### Statistical analysis

**Figure 3.**
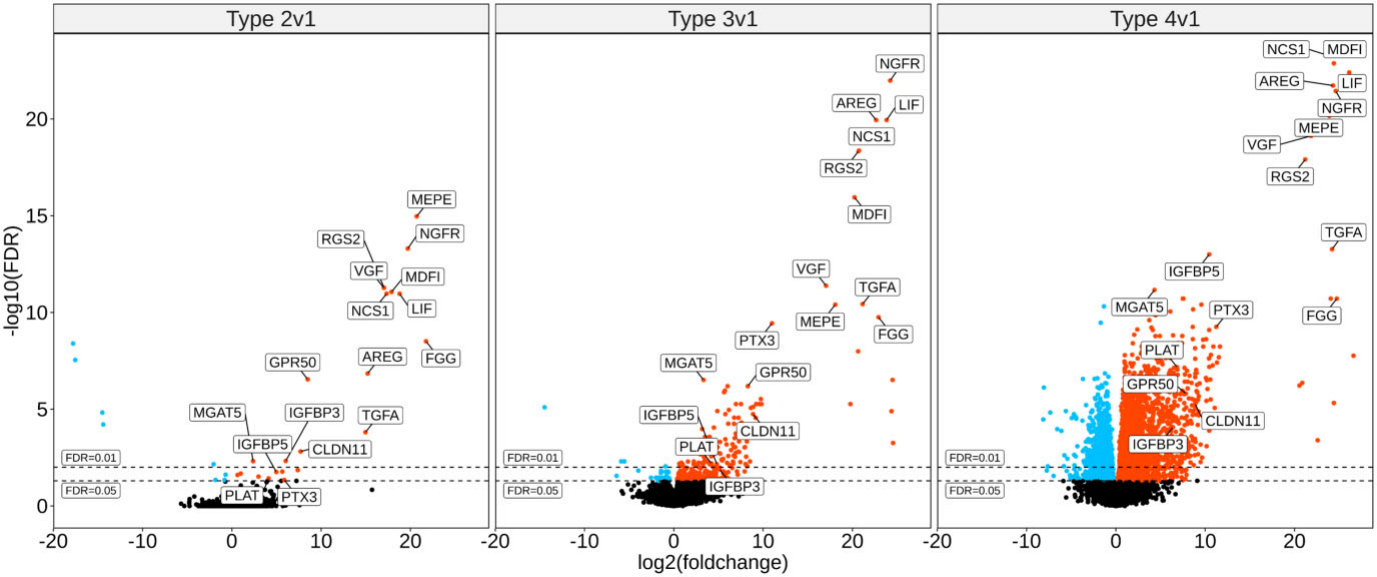
**Volcano plots comparing cumulus cell differentially expressed genes for Type 2, 3, and 4 versus 1 ovulatory-size follicles in the small ovarian reserve heifer.** Volcano plots of cumulus cell transcriptome data noting locations of the 17 DEGs described in this analysis. Data are from the previously published transcriptome study (Clark *et al.*, 2022b). The panels show data for the comparisons noted above each panel (left to right, Type 2, 3, and 4 versus Type 1). Each panel plots the Log_2_(foldchange) along the *x* axis, and the −Log_10_ (FDR) from the DESeq2 output from our previous study (Clark *et al.*, 2022b). Horizontal dashed lines denote two FDR thresholds: 0.01 and 0.05. Point color key: black = non-DEG; red = upregulated DEGs; blue = downregulated DEGs. The 17 DEGs common to all follicle types are indicated by their gene symbols. DEGs, differentially expressed genes; FDR, false discovery rate.

The R package DSeq2 (Love *et al.*, 2014) was used in our previous transcriptome study (Clark *et al.*, 2022b) to determine that 3288 genes in cumulus cells were significantly (false discovery rate (FDR) <0.01, except where noted as FDR < 0.05 in Table 1) DEGs when Type 2, 3, and 4 ovulatory-size follicles were compared with Type 1 ovulatory-size follicles. In the present study, we manually interrogated each of these Ex-cpFSH-induced 3288 cumulus cell DEGs (Clark *et al.*, 2022a) to determine whether any of the cumulus DEGs were common to all ovulatory-size follicles independent of their individual phenotypes. For statistical comparisons where zero expression values are obtained for genes in some samples (Fig. 3), DESeq2 uses a maximum likelihood estimate to assign very small values less than one to those entries. The maximum likelihood estimate reflects the maximum likelihood of detection with a small number of additional reads counted.

**Table 1:** Average fragments per kilobase per million^§^ mapped reads values for differential expression of cumulus genes critical for regulation of cumulus function and oocyte maturation in Type 1 compared with Ex-cpFSH-induced Types 2, 3, and 4 ovulatory-size follicles.

			Ovulatory-size follicle types			
Gene symbol	Gene name	Functions	Control	Ex-cpFSH		
			Type 1 (n = 7)	Type 2 (n = 6)	Type 3 (n = 6)	Type 4 (n = 5)
*AREG*	Amphiregulin	ORM (Procházka *et al.*, 2011; Assidi *et al.*, 2013), CE (Procházka *et al.*, 2011; Assidi *et al.*, 2013)	0	0.1	25	77
*CLDN11*	Claudin 11	JXN (Gow *et al.*, 1999; Elkouby-Naor and Ben-Yosef, 2010; Zhang *et al.*, 2018)	0.5	100	226	225
*FGG*	Fibrinogen G Gamma	Cl (Simurda *et al.*, 2020)	0	12	28	98
*GPR50*	G protein-coupled receptor 50	G (Assidi *et al.*, 2013; Vander Ark *et al.*, 2018), ORM (Chen *et al.*, 2022)	7	2474	2133	1564
*IGFBP3*	Insulin-like growth factor binding protein 3	G (Yoshimura *et al.*, 1996), ORM (Yoshimura *et al.*, 1996), A (Chun *et al.*, 1994)	3	189	102	243*
*IGFBP5*	Insulin-like growth factor binding protein 5	G (Onoda *et al.*, 1995), A (Monget *et al.*, 1998; Canty *et al.*, 2006; Hatzirodos *et al.*, 2014; Mazerbourg and Monget, 2018)	15*	479	429	21367
*LIF*	Leukemia-inhibitory factor	ORM (Dang-Nguyen *et al.*, 2014; Mo *et al.*, 2014), CE (De Matos *et al.*, 2008)	0	2	55	270
*MDFI*	MyoD family inhibitor	O (Espey, 1978) (Martin *et al.*, 1983)	0	0.8	5	77
*MEPE*	Matrix extracellular phosphoglycoprotein	ECM (Irving-Rodgers and Rodgers, 2005; Cho *et al.*, 2009; Nagyová *et al.*, 2021)	0	7	1	63
*MGAT5*	Alpha-1,6-manosyl-glycoprotein beta-1,6-acetylglucosaminyltransferase	CE (Lo *et al.*, 2019), ECM (Lo *et al.*, 2019)	56	285	558	1111
*NCS1*	Neuronal calcium sensor 1	Ca (Rousset *et al.*, 2003; Boeckel and Ehrlich, 2018)	0	0.6	7	60
*NGFR*	Nerve growth factor receptor	JXN (Zhai *et al.*, 2018), A (Zhou *et al.*, 2016)	0	3	74	97
*PLAT*	Plasminogen Activator, Tissue	ECM (Rifkin, 1992), CE (Yu, 2019), ORM (Yu, 2019)	39*	673	727	4460
*PTX3*	Pentraxin 3	CE (Varani *et al.*, 2002), ECM (Baranova *et al.*, 2014; Nagyova *et al.*, 2016; Fan and de Sousa Lope, 2021)	2	122*	4232	5030
*RGS2*	Regulator of G protein signaling 2	G (Landomiel *et al.*, 2014), Ca (Bernhardt *et al.*, 2015), A (Dong *et al.*, 2017)	0	6	71	102
*TGFα*	Transforming growth factor alpha	G (Legault *et al.*, 1999), ORM (Brucker *et al.*, 1991; Mito *et al.*, 2013), CE (Kobayashi *et al.*, 1994; Assidi *et al.*, 2013)	0	0.1	9	73
*VGF*	VGF nerve growth factor inducible	G (Hahm *et al.*, 1999; Aguilar *et al.*, 2013; Choi *et al.*, 2016), Ca (Severini *et al.*, 2007; Bresciani *et al.*, 2019)	0	7	7	193

^**§**^FPKM values for cumulus DEGs were determined for the Type 1, 2, 3, and 4 ovulatory-size follicles excised from small ovarian reserve Holstein heifers subjected to ovarian stimulation with IS-cpFSH (control) or Ex-cpFSH doses. The number in parentheses under each follicle type indicates the number of individual ovulatory-size follicles and individual animals. Zero = undetectable values. Box Whisker Plots of these results are depicted in Fig. 4. Type 1 ovulatory-size follicles differed (*P* < 0.01, *P < 0.05) from Type 2, 3, or 4 follicles for each gene based on DSeq2 analysis. A = apoptosis; Ca = calcium movement; CE = cumulus expansion; Cl = clotting regulation; ECM = extracellular matrix; G = Gonadotrophin action/secretion; JXN = tight junction and cell communication; O = ovulation; ORM = oocyte resumption of meiosis. FPKM, fragments per kilobase per million mapped reads; DEGs, differentially expressed genes; IS-cpFSH, industry-standard commercial FSH-enriched porcine pituitary preparation; Ex-cpFSH, excessive commercial FSH-enriched porcine pituitary preparation.

We conducted three experiments (Exp) in the present study to determine if ovarian stimulation of the SORH model with Ex-cpFSH dysregulates cumulus function and oocyte nuclear maturation. Prior to data collection, a power analysis was conducted to determine the minimum number of animals and COCs per animal required to detect effects at a significance level of *P* ≤ 0.05 at a power level of 0.80 using a balanced crossover design (SAS Inc. 2013). Analysis determined that a minimum of five animals per dose (10 animals total) and a minimum of three to five COCs per animal would be sufficient to meet these criteria.

To minimize animal numbers and confounding variables, a balanced crossover design was used. Each heifer acted as its own control, and each heifer was subjected to ovarian stimulation twice with a different cpFSH dose [70 IU (40 mg/2 ml) or 210 IU (120 mg/6 ml)] and with a different cpFSH dose sequence (e.g. 70, 210 IU versus 210, 70 IU) at the first and second ovarian stimulation regimen (Karl *et al.*, 2021). One heifer in Exp 2 was hyper-responsive to both doses of cpFSH (70 and 210 IU; 60 and 70 ovulatory-size follicles, respectively). When normality and distribution were evaluated statistically, that heifer was a statistical outlier and thus removed from further analysis (SAS Inc., 2019). In Exp 3, several heifers from each cpFSH dose group were removed from statistical analysis because oocyte retrieval resulted in <3 COCs (control, one heifer removed; Ex-cpFSH, four heifers removed), as explained above.

The responses for Exp 1–3 were expressed as binomial proportions using total number of COCs analyzed as the denominator. The model statement for logistic regression analysis included dose of cpFSH, COC morphology, oocyte nuclear stage of maturation, and the associated interaction between these variables. Estimates were reported as the mean (±SEM) proportion of ovulatory follicles per heifer with comCOCs, expCOCs or denuded oocytes, or proportion of ovulatory-size follicles per heifer with comCOCs or expCOCs at GV, GVBD, MI, MII, or with degenerated oocytes. Unless specified otherwise, all statistical analyses were performed using Statistical Analysis System (SAS 9.4 Institute, Cary, NC, USA) PROC GLIMMIX and PROC LOGISTIC (SAS Inc., 2015, 2019). When using PROC LOGISTIC, Firth’s Penalized Likelihood was also used for analyzing the stages of oocyte nuclear maturation data from each Exp to mitigate non-convergence caused by the presence of all-zero responses for a particular treatment within the dataset (Karabon, 2020). The cpFSH dose effects were considered significant if *P *≤* *0.05.

## Results

### Ex-cpFSH induces across all follicle phenotypes 17 DEGs that have predicted roles in regulation of cumulus cell function and oocyte maturation

**Figure 4.**
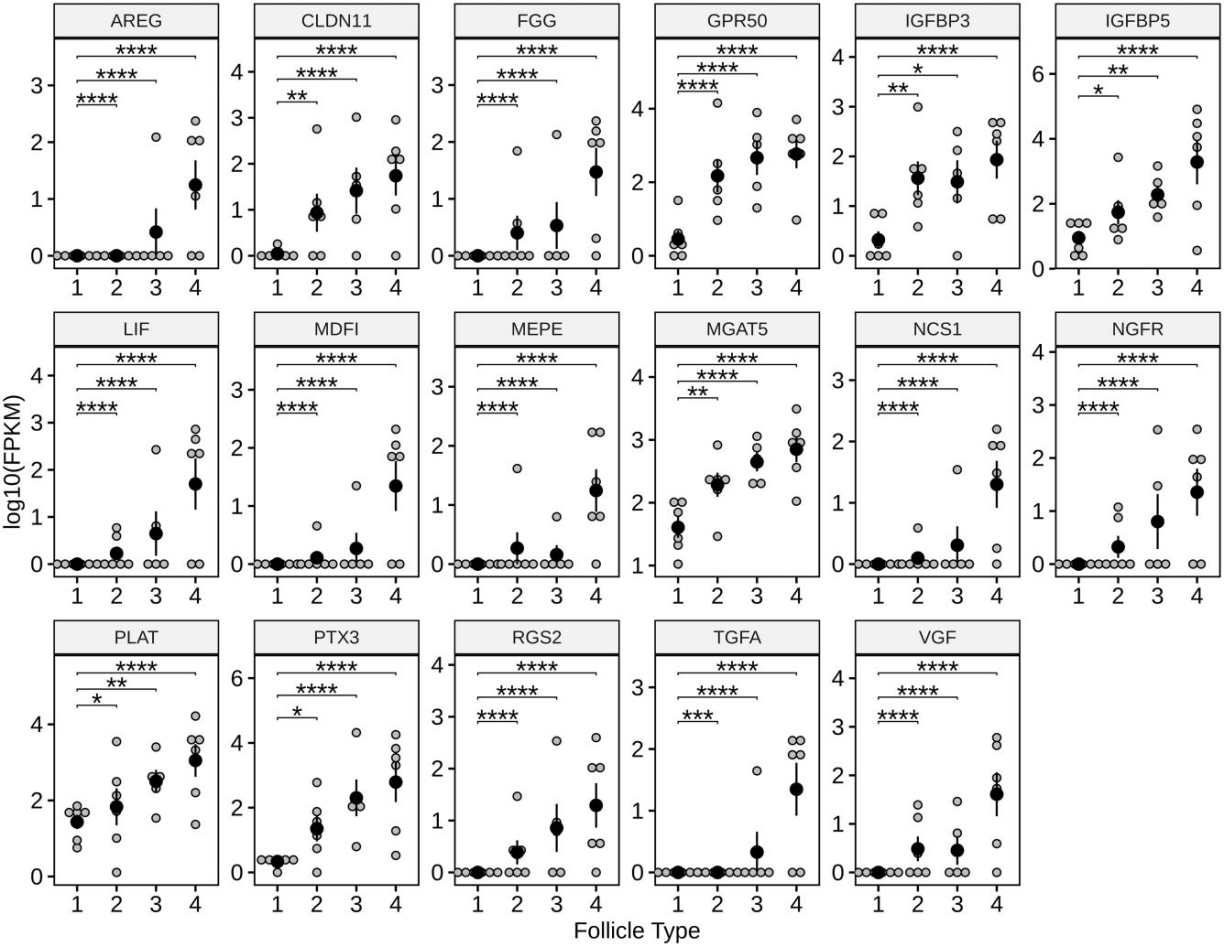
**Expression of cumulus cell genes critical for regulation of cumulus function and oocyte maturation in Type 1 compared with excessive cpFSH-induced Types 2, 3, and 4 ovulatory-size follicles.** Small ovarian reserve Holstein heifers in our previous study (Clark *et al.*, 2022b) were subjected to ovarian stimulation with IS-cpFSH (control) or Ex-cpFSH doses and cumulus cells removed from the different types ovulatory-size follicles and subjected to RNAseq and bioinformatic analyses (Table 1 footnote). Types 1, 2, 3, and 4 ovulatory-size follicles are depicted along the *x* axis. Data points within each panel reflect mean expression values for replicates of each gene based on FPKM (*y* axis) determined in our previous study (Clark *et al.*, 2022b). Analysis of our previously published data identified 17 shared cumulus DEGs (FDR <0.05 to FDR <0.01) common to all the Type 2, 3, and 4 ovulatory-size follicles. These 17 Ex-cpFSH-induced cumulus cell DEGs all have well-established roles in modulation of FSH action and cumulus cell function including induction of cumulus cell expansion and regulation of oocyte maturation (Table 1). Gene symbols are depicted at the top of each panel (names and functions in Table 1). Within each panel, the large black circles denote mean FPKM values, small gray circles denote each sample, and vertical lines denote SEM. Based on statistical analysis results (using DSeq2 software) from our previous study (Clark *et al.*, 2022b), the horizontal lines at the top of each panel represent comparisons between Type 1 versus 2, 3, or 4 ovulatory-size follicles. Asterisks above each horizontal line indicate if the FDR values for each mean comparison differed significantly (*P ≤ 0.05, **P ≤ 0.01, ***P ≤ 0.001, ****P ≤ 0.0001). IS-cpFSH, industry-standard commercial FSH-enriched porcine pituitary preparation; Ex-cpFSH, excessive commercial FSH-enriched porcine pituitary preparation; FPKM, fragments per kilobase per million mapped reads; DEG, differentially expressed gene; FDR, false discovery rate.

This analysis of our previously published data yielded 17 shared cumulus DEGs common to Type 2, 3, and 4 ovulatory-size follicles as depicted in Volcano (Fig. 3) and Box Whisker Plots (Fig. 4). These 17 Ex-cpFSH-induced cumulus cell DEGs all have well-established roles in modulation of FSH action and cumulus cell function, including induction of cumulus cell expansion and regulation of oocyte maturation as indicated in Table 1.

### Ex-cpFSH did not increase number of ovulatory-size follicles or COC recovery

The numbers of ovulatory-size follicles for each heifer were counted, and COCs were recovered 12 h after the last cpFSH injection in Exp 1, and 3 and 24 h after the ovulatory hCG stimulus in Exp 2. The number of ovulatory-size follicles, number of aspirated ovulatory-size follicles, number of COCs recovered, and COC recovery rate were similar (*P* > 0.05) between heifers treated with the different cpFSH doses (Table 2).

**Table 2:** Impact of ovarian stimulation of small ovarian reserve heifers with control or Ex-cpFSH doses on number of ovulatory-size follicles and cumulus–oocyte complex recovery.

	Exp 1		Exp 2		Exp 3	
	Control	Ex-cpFSH	Control	Ex-cpFSH	Control	Ex-cpFSH
**Number of ovulatory-size follicles (≥10 mm)**	19 ± 5 (6–56)	31 ± 6 (9–59)	21 ± 4 (6–60)	26 ± 4 (9–70)	17 ± 3 (5–46)	19 ± 2 (3–31)
**Number of aspirated follicles**	16 ± 4 (5–41)	24 ± 4 (8–44)	17 ± 2 (5–32)	20 ± 3 (8–50)	18 ± 3 (13–56)	18 ± 2 (19–45)
**Number of COCs recovered by OR**	10 ± 3 (2–28)	13 ± 2 (5–26)	12 ± 2 (2–27)	13 ± 2 (2–31)	10 ± 2 (5–41)	11 ± 1 (9–28)
**COC recovery rate**	58% ± 5% (29–90%)	53% ± 6% (22–85%)	58% ± 3% (33–100%)	63% ± 5% (25–87%)	55% ± 5% (13–100%)	56% ± 5% (10–82%)

The small ovarian reserve Holstein heifers in Exp 1 (n = 10/dose) and 3 (n = 18/dose) were subjected to OR to recover COCs 12 h after the last cpFSH injection while the heifers in Exp 2 (n = 16/dose) were subjected to an ovulatory hCG stimulus 12 h after the last cpFSH injection, and COCs were recovered by OR 24 h after the hCG injection but before ovulation. The number of ovulatory-size follicles was determined at the time of OR. Recovery rate = number of COCs recovered by OR divided by the number of aspirated follicles. Data are expressed as mean ± SEM per heifer with the range of the data collected in parentheses. Based on Type III ANOVA analysis, there were no statistical differences between means. Exp, experiment; IS-cpFSH, industry-standard commercial FSH-enriched porcine pituitary preparation; Ex-cpFSH, excessive commercial FSH-enriched porcine pituitary preparation; OR: oocyte retrieval; COCs, cumulus–oocyte complexes.

### Ex-cpFSH induced premature cumulus expansion and resumption of meiosis prior to an ovulatory hCG stimulus

The COCs recovered by oocyte retrieval 12 h after the last cpFSH injection (Exps 1 and 3) were classified as comCOCs, expCOCs, or denuded. Only comCOCs or denuded oocytes were recovered by oocyte retrieval in the controls. In contrast, comCOCs, expCOCs, and denuded oocytes were recovered from heifers treated with Ex-cpFSH doses (Table 3, Exps 1 and 3). The proportion of ovulatory-size follicles with expCOCs per heifer was higher (*P *<* *0.0001), while the proportion with comCOCs was lower (*P *<* *0.01), in the Ex-cpFSH treated heifers compared with controls for Exps 1 and 3 (Table 3).

**Table 3:** Impact of ovarian stimulation of small ovarian reserve heifers with control or Ex-cpFSH doses on morphology of cumulus–oocyte complexes.

Morphological classification of COCs	Exp 1		Exp 2		Exp 3	
	Control	Ex-cpFSH	Control	Ex-cpFSH	Control	Ex-cpFSH
**comCOC**	93% ± 3% (75–100%)	77% ± 4%** (58–100%)	46% ± 9% (0–100%)	72% ± 5%** (27–100%)	91% ± 3% (0–94%)	65% ±5%**** (40–100%)
**expCOC**	0% ± 0.4% (0–4%)	22% ± 5%**** (0–42%)	45% ± 8% (0–100%)	24% ± 4%* (0–39%)	3% ± 2% (0–29%)	32% ±5%**** (0–82%)
**Denuded**	3% ± 3% (0–25%)	0.7% ± 1% (0–7%)	7% ± 2% (0–25%)	4% ± 1% (0–17%)	6% ± 3% (0–44%)	3% ± 1% (0–18%)

The small ovarian reserve Holstein heifers in Exp 1 (n = 10/dose) and 3 (n = 18/dose) were subjected to OR to recover COCs 12 h after the last cpFSH injection while the heifers in Exp 2 (n = 16/dose) were subjected to an ovulatory hCG stimulus 12 h after the last cpFSH injection, and COCs were recovered by OR 24 h after the hCG injection but before ovulation. Proportions per column may not total 100% because some COCs or oocytes were unidentifiable or lost during histological processing to assess nuclear maturation. Data are expressed as mean ± SEM for proportions of ovulatory-size follicles per heifer with COCs classified as comCOC, expCOC, or denuded with the range of the data collected in parentheses.

* *P* ≤ 0.05,

** *P* ≤ 0.01,

**** *P* ≤ 0.0001 denote statistical differences between means within each experiment based on Type III ANOVA analysis. Exp, experiment; IS-cpFSH, industry-standard commercial FSH-enriched porcine pituitary preparation; Ex-cpFSH, excessive commercial FSH-enriched porcine pituitary preparation; OR: oocyte retrieval; COCs, cumulus–oocyte complexes; comCOC, compact cumulus–oocyte complex; expCOC, expanded cumulus–oocyte complex.

The impact of the Ex-cpFSH treatment on nuclear maturation prior to the ovulatory hCG stimulus was only examined in Exp 1. Results show that 93% of ovulatory-size follicles in controls and 77% in the Ex-cpFSH treated heifers had comCOCs (Table 3, Exp 1). Of the comCOCs, 77% in controls and 97% in the Ex-cpFSH treated heifers were at the GV stage (Table 4). Although none of the comCOCs had resumed meiosis in controls, a very low proportion (2%) of the ovulatory-size follicles for the heifers treated with the Ex-cpFSH dose had comCOCs that had resumed meiosis (MI, MII, Table 4). However, the proportions of ovulatory-size follicles with comCOCs at MI or MII for the Ex-cpFSH treated heifers did not differ (*P* > 0.05) from controls (Table 4). In contrast to controls, 22% of the ovulatory-size follicles had expCOCs (Table 3, Exp 1), and 49% of the expCOCs had resumed meiosis (GVBD + MI + MII) in the Ex-cpFSH treated heifers (Table 4). The remainder of the expCOCs in the Ex-cpFSH treated heifers were at the GV stage, had oocytes classified as degenerated or fragmented, or were lost during processing.

**Table 4:** Impact of ovarian stimulation of small ovarian reserve heifers with control or Ex-cpFSH doses on stage of nuclear maturation for compact cumulus–oocyte complexes or expanded cumulus–oocyte complexes.

	Exp 1		
	Control	Ex-cpFSH	
	comCOC	comCOC	expCOC
**Number of oocytes evaluated per heifer**	9 ± 3 (1–27)	7 ± 1 (1–14)	3 ± 1 (0–9)
**Stage of nuclear maturation**			
**GV**	77% ± 8% (30–100%)	97% ± 3% (75–100%)	19%±10%**** (0–100%)
** GVBD**	0% ± 0%	0% ± 0%	1% ± 1% (0–11%)
** MI**	0% ± 0%	1% ± 3% (0–13%)	40% ± 13%** (0–100%)
** MII**	0% ± 0%	1% ± 1% (0–13%)	8% ± 5% (0–7%)
** Degenerated**	2% ± 2% (0–14%)	0.8% ± 1% (0–33%)	8% ± 4% (0–33%)

Small ovarian reserve Holstein heifers (n = 10/dose) were subjected to ovarian stimulation with IS-cpFSH (control) or Ex-cpFSH doses then subjected to OR to recover COCs 12 h after the last cpFSH injection. Proportions per column may not total 100% because some COCs or oocytes were unidentifiable or lost during histological processing to assess nuclear maturation. Data are expressed as mean ± SEM for proportion of ovulatory-size follicles with comCOCs or expCOCs at each nuclear maturation stage per heifer with the range of the data collected in parentheses.

** *P* ≤ 0.01,

**** *P* ≤ 0.0001 denote statistical differences between means within cpFSH dose based on Type III ANOVA analysis. IS-cpFSH, industry-standard commercial FSH-enriched porcine pituitary preparation; Ex-cpFSH, excessive commercial FSH-enriched porcine pituitary preparation; OR: oocyte retrieval; COCs, cumulus–oocyte complexes; comCOC, compact cumulus–oocyte complex; expCOC, expanded cumulus–oocyte complex; GV, germinal vesicle, GVBD, germinal vesicle breakdown; MI, metaphase I; MII, metaphase II.

### Ex-cpFSH blocked cumulus expansion and resumption of meiosis in response to an ovulatory hCG stimulus

In response to the ovulatory hCG stimulus, the proportion of ovulatory-size follicles per heifer with comCOCs was higher (*P* < 0.01) while the proportion with expCOCs was lower (*P *<* *0.05) for the heifers treated with Ex-cpFSH doses compared with controls (Table 3, Exp 2). Surprisingly, although prematurely expCOCs (as observed in Exps 1, 3; see Clark *et al.* (2022a,b) for images) could not be distinguished morphologically from expCOCs after the ovulatory hCG stimulus, the proportion of ovulatory-size follicles with expCOCs was nearly identical to the results for the Ex-cpFSH treated heifers in Exps 1, 3 prior to an ovulatory hCG stimulus (Table 3).

We observed that 71% of the expCOCs in controls and 65% of the expCOCs in the Ex-cpFSH treated heifers had resumed meiosis (GVBD + MI + MII) after the ovulatory hCG stimulus (Table 5). The Ex-cpFSH treated heifers had a higher (*P* < 0.01) proportion of ovulatory-size follicles with expCOCs at MI but a lower (*P* < 0.001) proportion at MII, compared with controls. In addition, the Ex-cpFSH treated heifers had a lower (*P* < 0.0001) proportion of ovulatory-size follicles with expCOCs at GV but a higher proportion at MI (*P* < 0.01) and MII (*P* < 0.001) compared with the comCOCs from the Ex-cpFSH treated heifers (Table 5).

**Table 5:** Impact of an ovulatory hCG stimulus following ovarian stimulation of small ovarian reserve heifers with control or Ex-cpFSH doses on stage of nuclear maturation for compact cumulus–oocyte complexes or expanded cumulus–oocyte complexes.

	Exp 2			
	Control		Ex-cpFSH	
	comCOC	expCOC	comCOC	expCOC
**Number of oocytes evaluated per heifer**	2 ± 0.5 (0–6)	4 ± 1 (0–14)	4 ± 0.8 (1–13)	3 ± 0.7 (0–9)
**Stage of nuclear maturation**				
** GV**	68% ± 12% (0–100%)	4% ± 3%*** (0–40%)	86%± 3%^++^ (50–100%)	11% ± 5%**** (0–50%)
** GVBD**	1% ± 1% (0–17%)	2% ± 2% (0–25%)	4% ± 3% (0–50%)	0.7% ± 1% (0–11%)
** MI**	0% ± 0%	12% ± 6%* (0–75%)	1% ± 1% (0–17%)	30% ± 10%** (0–100%)
** MII**	0% ± 0%	57%± 12%**** (0–100%)	0.7% ± 1% (0–11%)	34%±8%^++++^*** (0–100%)
** Degenerated**	0% ± 0%	0% ± 0%	7% ± 6% (0–11%)	7% ± 3% (0–50%)

Small ovarian reserve Holstein heifers were subjected to ovarian stimulation with IS-cpFSH (control, n = 15) or Ex-cpFSH (n = 16) doses and then subjected to an ovulatory hCG stimulus 12 h after the last cpFSH injection, and COCs were recovered by OR 24 h after the hCG injection but before ovulation. Proportions per column may not total 100% because some COCs or oocytes were unidentifiable or lost during histological processing to assess nuclear maturation. Proportions per column may not total 100% because some COCs or oocytes were unidentifiable or lost during histological processing to assess nuclear maturation. Data are expressed as means ± SEM for proportion of ovulatory-size follicles with COCs at each nuclear classification per heifer with the range of the data collected in parentheses.

* *P* ≤ 0.05,

** *P* ≤ 0.01,

*** *P* ≤ 0.001.

**** *P* ≤ 0.0001 denote statistical differences between means within dose whereas plus. (^++++^*P* ≤ 0.0001) denote statistical differences between means but within COC classification across cpFSH doses based on Type III ANOVA analysis. IS-cpFSH, industry-standard commercial FSH-enriched porcine pituitary preparation; Ex-cpFSH, excessive commercial FSH-enriched porcine pituitary preparation; OR, oocyte retrieval; COCs, cumulus–oocyte complexes; comCOC, compact cumulus–oocyte complex; expCOC, expanded cumulus–oocyte complex; GV, germinal vesicle, GVBD, germinal vesicle breakdown; MI, metaphase I; MII, metaphase II.

After the ovulatory hCG stimulus, we unexpectedly observed that 46% of the ovulatory-size follicles from controls and 72% of the ovulatory-size follicles from heifers treated with the Ex-cpFSH doses had comCOCs (Table 3). Moreover, only 1% of the ovulatory-size follicles with the comCOCs had resumed meiosis in the control compared with ∼6% in the Ex-cpFSH treated heifers (Table 5). Like Exp 1, however, the proportions of ovulatory-size follicles with comCOCs at GVBD, MI, or MII were similar between treatment groups (Table 5).

### Ex-cpFSH-induced prematurely expCOCs responded poorly to IVF

The proportion of expCOCs that cleaved and formed blastocysts or hatched blastocysts after IVF was much lower (*P* < 0.05 to *P* < 0.0001) compared with IVF results for IVM-matured comCOCs (Table 6). The very poor cleavage results for expCOCs are unlikely caused by the small number of COCs per IVF group, especially since 12 of 20 (60%) singlets and 38 of 52 (73%) pairs of comCOCs subjected to IVF cleaved by Day 2 of culture.

**Table 6:** IVF results for bovine oocytes with prematurely expanded or compact layers of cumulus cells.

Exp 3				
COC morphology	N	Cleaved	Blastocyst	Hatched Blastocyst
**comCOC**	182	124 (68%)	51 (28%)	22 (12%)
**expCOC**	37	2 (5.4%)****	0 (0%)***	0 (0%)*

The comCOC were recovered from follicles of ovaries obtained at a local abattoir and subjected to IVM and IVF. Prematurely expCOC were obtained from Ex-cpFSH treated heifers (n = 8) 12 h after the last cpFSH injection but prior to an ovulatory hCG stimulus and subjected to IVF (using sperm from the same bull) without IVM. Data are represented as the number of fertilized oocytes from comCOC or expCOC subjected to IVF that cleaved, formed blastocysts, or hatched blastocysts divided by total number of comCOC or expCOC subjected to IVF with the ratio in parentheses.

* *P* ≤ 0.05,

*** *P* ≤ 0.001,

**** *P* ≤ 0.0001 denote significant difference between ratios when comCOC were compared with expCOC based on Type III ANOVA analysis. comCOC, compact cumulus–oocyte complex; expCOC, expanded cumulus–oocyte complex; Ex-cpFSH, excessive commercial FSH-enriched porcine pituitary preparation; N, number of comCOC or expCOC.

### Ex-cpFSH reduced the capacity of comCOCs and expCOCs recovered prior to an ovulatory hCG stimulus to resume meiosis during IVM

The vast majority (91%) of the ovulatory-size follicle with comCOCs resumed meiosis during IVM (Table 7). Nevertheless, the proportion of ovulatory-size follicles with comCOCs that progressed to MII after IVM was lower (*P* < 0.01) for the Ex-cpFSH treated heifers compared with controls (Table 7). None of the controls had expCOCs as observed in Exp 1. In contrast, 32% of the ovulatory-size follicles recovered 12 h after the last cpFSH injection (prior to the ovulatory hCG stimulus) had expCOCs (Table 3, Exp 3). However, only 38% of the premature expCOCs resumed meiosis and reached MII during IVM, while 55% were classified as degenerated (Table 6). For the Ex-cpFSH treated heifers, the proportion of ovulatory-size follicles with expCOCs that reached MII during IVM was lower (*P* < 0.0001) while the proportion classified as degenerated was higher (*P* < 0.05) compared with comCOCs subjected to IVM (Table 7).

**Table 7:** Impact of ovarian stimulation of small ovarian reserve heifers with control or Ex-cpFSH doses on stage of nuclear maturation of compact cumulus–oocyte complexes or expanded cumulus–oocyte complexes after IVM.

	Exp 3		
	Control	Ex-cpFSH	
	comCOC	comCOC	expCOC
**Number of oocytes evaluated per heifer**	9 ± 2 (2–33)	7 ± 1 (2–20)	4 ± 0.8 (0–11)
**Stage of nuclear maturation after IVM**			
** GV**	8% ± 3% (0–43%)	9% ± 2% (0–25%)	8% ± 6% (0–50%)
** GVBD**	6% ± 2% (0–25%)	5% ± 2% (0–25%)	0% ± 0%
** MI**	3% ± 2% (0–33%)	6% ± 2% (0–25%)	0% ± 0%
** MII**	82% ± 4% (50–100%)	70%±6%^**++**^ (17–100%)	38%±16%**** (0–100%)
** Degenerated**	1% ± 1% (0–9%)	10% ± 4% (0–50%)	55% ± 14%* (0–100%)

COCs were recovered from small ovarian reserve Holstein heifers subjected to ovarian stimulation with IS-cpFSH (control, n = 17 heifers) or Ex-cpFSH (n = 14) doses and IVM for 22 h. Proportions per column may not total 100% because some COCs or oocytes were unidentifiable or lost during histological processing to assess nuclear maturation. Data are expressed as mean ± SEM for proportion of ovulatory-size follicles with comCOCs or expCOCs at each nuclear maturation stage per heifer with the range of the data collected in parentheses.

* *P* ≤ 0.05,

**** *P* ≤ 0.0001 denote statistical differences between means within cpFSH dose whereas pluses. (^++^*P* ≤ 0.01) denote statistical difference between means across dose but within COC classification across cpFSH doses based on Type III ANOVA analysis. COCs, cumulus–oocyte complexes; IS-cpFSH, industry-standard commercial FSH-enriched porcine pituitary preparation; Ex-cpFSH, excessive commercial FSH-enriched porcine pituitary preparation; comCOC, compact cumulus–oocyte complex; expCOC, expanded cumulus–oocyte complex; GV, germinal vesicle, GVBD, germinal vesicle breakdown; MI, metaphase I; MII, metaphase II.

## Discussion

The most significant findings in the present study are that Ex-cpFSH doses during the ovarian stimulation of the SORH model: increase expression of 17 genes in cumulus cells with well-established roles in cumulus expansion, function, and regulation of resumption of meiosis in all ovulatory-size follicles prior to an ovulatory hCG stimulus; induce premature (prior to ovulatory hCG stimulus) expansion of the COC and resumption of meiosis in a moderate proportion of ovulatory-size follicles; impair the capacity of prematurely expCOCs to undergo IVF and resume meiosis during IVM; and reduce responsiveness of comCOCs to an ovulatory hCG stimulus. Taken together, these observations in the SORH biomedical model support the conclusion that excessive FSH doses during ovarian stimulation dysregulate cumulus cell function thereby impairing oocyte quality, contributing to oocyte wastage, and diminishing IVF success and ART outcomes.

The present study extends our previous transcriptome analysis (Clark *et al.*, 2022a,b) by showing that the Ex-cpFSH treatment induces differential expression of a small number of genes (17 of the 3288) in cumulus cells of all the ovulatory-size follicles independent of their extreme phenotypic differences. Many of these cumulus cell DEGs are regulated directly by FSH (*AREG, IGFBP-3, IGFBP-5, PTX3, RGS2, TGFα, PLAT, FGG*) in a variety of species. Moreover, most of these DEGs have well-established critical roles in regulation of gonadotrophin action (*GPR50, IGFBP-3, IGFBP-5, RGS2*) or secretion (*VGF*) and apoptosis (*IGFBP-3, IGFBP-5, RGS-2*), which could directly dysregulate cumulus cell function. Although *IGFBP-3, IGFBP-5*, and *RGS-2* are pro-apoptotic, results of ingenuity pathway analysis in our previous transcriptome study (Clark *et al.*, 2022b) using a robust, high-quality data set did not indicate activation or inhibition of apoptosis. In addition, several of the cumulus cell DEGs identified in the present study are well-established regulators of cumulus expansion (*AREG, LIF, MGAT5, MEPE, PTX3, TGFα, PLAT*) and resumption of meiosis (*AREG, IGFBP-3, TGFα, PLAT*). However, other DEGs impact the extracellular matrix (*MEPE, MGAT5, PTX3*) and tight junctions and cell communication (*CLDN11, NGFR*), including calcium movement (*NCS1, RGS2, VGF*) in non-cumulus cell types that could also have an undiscovered yet critical role in cumulus function and capacity to regulate resumption of meiosis. Two DEGs serve other roles in cumulus cells (*FGG*, *MDFI*). FGG has a role in regulation of blood clotting (Simurda *et al.*, 2020) and *MDFI* inhibits myogenesis (Berkes and Tapscott, 2005) and may have a role in ovulation (Espey, 1978; Martin *et al.*, 1983). Thus, dysregulation of *FGG* and *MDFI* could contribute to the reduced ovulation rate observed for the heifers subjected to Ex-cpFSH during ovarian stimulation in our previous study (Karl *et al.*, 2021).

Taken together, the discovery of DEGs overexpressed in cumulus cells in response to Ex-cpFSH in all of the ovulatory-size follicles of Ex-cpFSH dosed animals provides new insight into potential mechanisms whereby excessive FSH action prior to an ovulatory hCG stimulus induces premature cumulus expansion, as observed in our previous (Clark *et al.*, 2022a,b) and present studies, and premature resumption of meiosis.

The 17 cumulus cell DEGs reported here for Ex-cpFSH treated heifers are induced prior to an ovulatory hCG stimulus and many of these genes are known to be involved in cumulus expansion. However, even though these DEGs are in COCs of all ovulatory-size follicles, we observed here and in our previous studies (Clark *et al.*, 2022a,b) that premature cumulus expansion did not occur in all ovulatory-size follicles. This disconnect very likely represents a potential lag between the Ex-cpFSH induced alterations in gene expression with initiation of the morphological changes causing cumulus expansion (e.g. extracellular matrix). In addition, differential responsiveness of ovulatory-size follicles to cpFSH, which may explain the high within animal variability observed in gene expression (Box Whisker Plots, Fig. 4), could also contribute to the disconnect between expression of genes involved in cumulus expansion and cumulus expansion.

In the Ex-cpFSH treated heifers, a high proportion (75%) of ovulatory-size follicle had expCOCs in our previous study (Clark *et al.*, 2022a,b) while only a moderate proportion (22% for Exp 1, 32% for Exp 3) of ovulatory-size follicles had expCOCs in the present study. We cannot rule out the possibility that animal variation or differences in cpFSH potency explain the differences in proportion of ovulatory-size follicles with expCOCs between our present and previous (Clark *et al.*, 2022a,b) studies. However, it is highly likely that expCOCs, often described as very sticky in a variety of species (Moulavi and Hosseini, 2018; Richards, 2018), are more fragile and difficult to remove from ovulatory-size follicles during oocyte retrieval than comCOCs. For example, oocyte retrieval damages COCs if the aspiration pressure is not optimal (Ward *et al.*, 2000; Rose, 2014; Renzini *et al.*, 2020), which could explain why denuded oocytes were observed after oocyte retrieval in the present study. Consistent with this possible explanation, denuded oocytes were not observed when COCs were removed from excised ovulatory-size follicles using gentle pressure on a syringe and needle, as in our previous study (Clark *et al.*, 2022a,b).

We recognize a larger number of the Ex-cpFSH induced prematurely expanded COCs (expCOCs) from additional cattle will be necessary to confirm the inferior IVF results compared with not only controls in the present study but also with typical IVF results following IVM or artificial insemination of cattle reported by others (Lonergan and Fair, 2016). Nevertheless, our findings indicate that the prematurely expanded COCs observed here are defective and have poor-quality oocytes. The precise reason for diminished oocyte competence is unknown but very likely arises owing to dysregulated progression through meiosis (Table 4). However, we also observed in the present study that nearly 38% of the expCOCs recovered by oocyte retrieval before an ovulatory hCG stimulus reached the MII stage during IVM. This finding implies that IVM of the prematurely expanded COCs could have improved the poor IVF results observed in the present study (Table 6), although this was not examined. Nevertheless, we observed that the moderate proportion of ovulatory-size follicles with prematurely expanded COCs developing to MII during IVM was highly variable amongst heifers, while a higher proportion had degenerated during IVM (Table 5). Thus, we expect that IVM of prematurely expanded COCs would only marginally improve IVF success for a limited number of heifers.

We established that Ex-cpFSH doses during ovarian stimulation of the SORH model reduced estradiol production (Karl *et al.*, 2021; Clark *et al.*, 2022a,b) and decreased the hCG-induced ovulation rate (Karl *et al.*, 2021). These findings indicated that the Ex-cpFSH doses during ovarian stimulation impair responsiveness of ovulatory-size follicles to an ovulatory hCG stimulus. If so, this could explain why both estradiol production and ovulation rate were reduced in our previous studies (Karl *et al.*, 2021). Results here further confirm the likelihood that Ex-cpFSH doses diminish the responsiveness of ovulatory-size follicles to an ovulatory hCG stimulus based on three comparisons. First, after the ovulatory hCG stimulus, the proportion of ovulatory-size follicles per heifer with comCOCs (Table 3, Exp 2) and proportion of comCOCs that remained at the GV stage (Table 5) were both higher in the Ex-cpFSH treated heifers compared with controls. Second, the proportions of ovulatory-size follicles per heifer treated with Ex-cpFSH doses that had comCOCs and expCOCs before (Table 3, Exp 1) and after (Table 3, Exp 2) the ovulatory hCG stimulus were nearly identical. Third, a higher proportion of the expCOCs remained at GV while a lower proportion reached MII in the Exp-cpFSH treated heifers compared with controls (Table 5) implying that the Exp-cpFSH doses impeded resumption and progression of nuclear maturation to MII. These combined findings, coupled with our previous results (Karl *et al.*, 2021; Clark *et al.*, 2022a,b), provide compelling evidence that the Ex-cpFSH treatment during ovarian stimulation (prior to the ovulatory hCG stimulus) blocks responsiveness of the ovulatory-size follicles to hCG thereby reducing availability of high-quality hCG-matured COCs for ART.

Previous studies in sheep show that typical ovarian stimulation regimens do not enhance responsiveness of all ovulatory-size follicles to FSH or LH (McNatty *et al.*, 2010). This observation may explain why, although lower than the Ex-cpFSH treated heifers, a relatively high proportion (46%) of ovulatory-size follicles per heifer in controls had comCOCs after the ovulatory hCG stimulus. While the precise reason is unclear, the results imply that ovarian stimulation, even with the industry-standard cpFSH doses (control), also hinders responsiveness to an ovulatory hCG stimulus. In support of this possibility, results of our previous study (Karl *et al.*, 2021) show that ∼20% of the ovulatory-size follicles per heifer developing in response to the control doses do not ovulate in response to an ovulatory hCG stimulus.

In summary, the results identified key Ex-cpFSH-induced DEGs in cumulus cells that may dysregulate cumulus function, enhance premature cumulus expansion, and impair oocyte quality in ovulatory-size follicles developing prior to an ovulatory hCG stimulus. In addition, the excessive FSH also induced inhibition of cumulus expansion and oocyte maturation post-hCG, and a reduced capacity of oocytes with prematurely expanded cumulus cells to undergo IVF or nuclear maturation during IVM. Although cumulus expansion is the hallmark for oocyte maturation in response to a preovulatory LH surge (Eppig, 1982; Ng *et al.*, 1999; Nevoral *et al.*, 2015; Robker *et al.*, 2018), our observations emphasize the risks of recovery of predominantly dysregulated COCs. Such compromised COCs are likely morphologically indistinguishable from high-quality hCG-matured COCs, which could diminish IVF success rates when excessive FSH doses are used during ovarian stimulation.

## Data Availability

Data generated in the article is available upon reasonable request to the corresponding author.
